# Geographical Variation in *Coxiella burnetii* Seroprevalence in Dairy Farms Located in South-Western Ethiopia: Understanding the Broader Community Risk

**DOI:** 10.3390/pathogens10060646

**Published:** 2021-05-23

**Authors:** Tatiana Proboste, Feyissa Begna Deressa, Yanjin Li, David Onafruo Kal, Benti Deressa Gelalcha, Ricardo J. Soares Magalhães

**Affiliations:** 1 UQ Spatial Epidemiology Laboratory, School of Veterinary Science, The University of Queensland, Gatton, QLD 4343, Australia; s4494989@student.uq.edu.au (Y.L.); r.magalhaes@uq.edu.au (R.J.S.M.); 2 School of Veterinary Medicine, College of Agriculture and Veterinary Medicine, Jimma University, Jimma P.O. Box 307, Ethiopia; feyissa.begna@gmail.com (F.B.D.); bentijc@gmail.com (B.D.G.); 3 College of Veterinary Science, Bahr El Ghazal University, Wau P.O. Box 10739, Sudan; donafruo@gmail.com; 4 Children’s Health and Environment Program, Child Health Research Centre, The University of Queensland, Brisbane, QLD 4101, Australia

**Keywords:** dairy cattle, *Coxiella burnetii*, predictive risk mapping, Q fever, seroprevalence, spatial modelling

## Abstract

Q fever is a zoonotic disease that is caused by *Coxiella burnetii* and leads to abortion and infertility in ruminants and debilitating disease in humans. Jimma zone, including Jimma town, located in the Oromia region of Ethiopia, was affected by an outbreak of abortions in ruminants related to Q fever infection between 2013 and 2015. This study aimed to investigate the geo-clustering of *C. burnetii* seroprevalence in dairy farms of Jimma town and identify the environmental risk factors associated with seroprevalence distribution. A total of 227 cattle were tested for antibodies against *C. burnetii* in 25 farms. We explored the clustering of *C. burnetii* seroprevalence using semivariograms. A geostatistical regression-based model was implemented to quantify the risk factors and to predict the geographical variation in *C. burnetii* seroprevalence at unsampled locations in Jimma town using OpenBugs. Our results demonstrated that the risk of exposure in dairy cattle varied across the landscape of Jimma town and was associated with environmental risk factors. The predictive map of *C. burnetii* seroprevalence showed that communities in the eastern part of Jimma town had the highest risk of exposure. Our results can inform community-level investigations of human seroprevalence in the high-risk areas to the east of Jimma.

## 1. Introduction

The febrile illness Q fever is caused by *Coxiella burnetii*, a Gram-negative intracellular bacterium [1]. Q fever is a zoonotic disease that is distributed worldwide, except in New Zealand. Q fever has been commonly described as an occupational disease, affecting veterinarians, farmers and slaughterhouse workers; however, it is not limited to these groups [2]. The main route for transmission to humans is through contaminated aerosol inhalations and the presentation of the disease can vary from acute symptoms, such as severe pneumonia, to chronic or an asymptomatic infection [1,3]. Domestic ruminants are considered the main reservoirs for *C. burnetii* bacteria and their clinical symptoms are characterised by reproductive disorders, such as abortion, stillbirth, retained placenta, infertility and birth weakness [4].

Coxiellosis has mainly been associated with the abortion of domestic ruminants, most commonly in ovines. The infections can occur due to close contact with infected placentas, body fluids or environmental contamination [5]. One common source of *C. burnetii* infection in a herd is the introduction and movement of domestic ruminants., which has been demonstrated in several outbreaks [6,7]. Environmental contamination plays a fundamental role in bacterium transmission, as *C. burnetii* that is attached to dust particles, can be dispersed by wind [8,9,10,11]. Even with the recognised importance of wind-borne dispersal in Q fever infections, there is a lack of empirical studies on the geographical dispersal of *C. burnetii* [12]. However, observational studies indicate that, on average, infection risk increases within a distance of 5 to 10 km of infected sources in rural areas, while in urban areas, outbreaks were associated with a distance between 2 to 4 km from contaminated sources [12]. 

In Africa, *C. burnetii* exposure is a common finding in a variety of species; however, the seroprevalence varies by species and location [13]. Studies on *C. burnetii* seroprevalence in cattle varied from 5% in Dakar, Senegal, to 55% in Zaria, Nigeria [13]. The seroprevalence of *C. burnetii* has previously been reported in pastoral livestock in south-east Ethiopia, Africa, with the highest seroprevalence found in camels (90%), followed by goats (54%) and cattle (31%) [14]. Between 2013 and 2015, the Jimma zone in the Oromia region, Ethiopia, was affected by an increasing trend of abortion episodes in ruminants over these years [15]. More than 11,487 cases were recorded, which included cattle, goats and sheep [16]. The overall seroprevalence in dairy cattle in the capital city of Jimma zone (Jimma town) was estimated to be close to 9%, with a higher seroprevalence in cattle at slaughterhouses than in cattle at dairy farms [15]. Despite the relatively high seroprevalence in livestock and the high cattle density in Jimma, the geographical variation in the distribution of *C. burnetii* exposure in the region is yet to be investigated. Rural communities in the Jimma zone are likely to be at a higher risk of exposure to *C. burnetii* due to the high density of cattle herds, extensive management systems, potential contact with other herds and associated socio-environmental conditions. Knowledge concerning the relative distribution of *C. burnetii* exposure in livestock is important for assessing the baseline risk to human communities in the vicinity of infected farms. 

To prevent and control future outbreaks, it is important to understand which areas may have a higher risk of infection and the role that environmental variables play in the risk of exposure. In the context of *C. burnetii* transmission, this approach is particularly important given that environmental conditions have been linked with human Q fever infections in several countries where livestock and human outbreaks have been reported in the same place [11,12,17]. Spatial epidemiological methods are useful for understanding the relationship between infection and covariables, such as land use and human population density, and to predict disease frequency indicators at unsampled locations. Model-based geostatistical and Bayesian approaches can be useful for disease control management by estimating the variation in disease risk and by highlighting areas of higher risk [18,19].

In this study, we aimed to identify the geographical distribution of *C. burnetii* seroprevalence in the herds of Jimma town, which contained the cattle with a higher risk of exposure, and quantify associated ecological risk factors. First, we explored the clustering of Q fever seroprevalence via spatial correlations; second, we developed a geostatistical regression model to quantify risk factors and predict the geographical risk of Q fever seroprevalence in Jimma town to uncover the likely exposure risk of communities in the vicinity of affected cattle herds.

## 2. Results

### 2.1. Spatial Variation of C. burnetii Seroprevalence and Analysis of the Clustering

**Figure 1:**
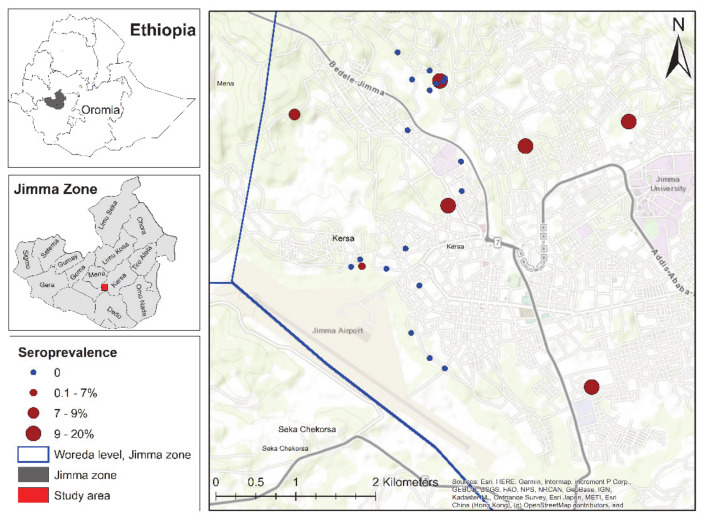
Spatial distribution of *Coxiella burnetii* seroprevalence related to the 25 dairy farms involved in the study. The study area (main map) is situated in the Jimma town in the Oromia region, Ethiopia.

**Figure 2:**
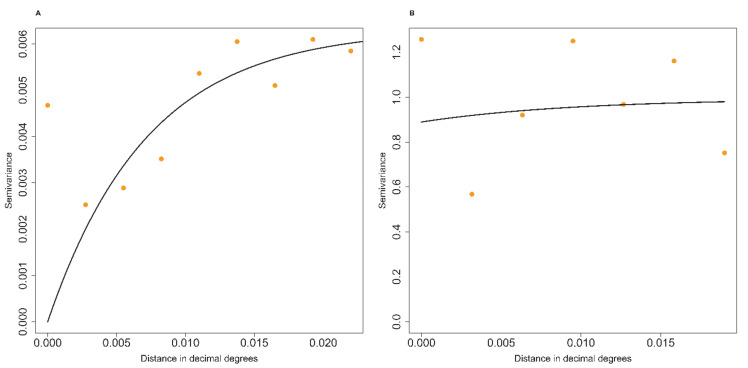
Geographical clustering of the *C. burnetii* seroprevalence in the 25 dairy farms involved in the study in Jimma town: (**A**) raw data semivariogram and (**B**) residual semivariogram. One decimal degree corresponds to approximately 111 km at the equator.

Our results indicated that three of the 25 farms with the highest seroprevalence were located in the eastern part of Jimma town (Figure 1). Moreover, the results of the semivariogram of the observed *C. burnetii* seroprevalence showed a tendency for clustering in the study area (Figure 2). The average cluster size of farm-level *C. burnetii* seroprevalence was approximately 800 m. 

The summary of the farm-level *C. burnetii* seroprevalence semivariogram (Table 1, Figure 2a) estimated partial sill = 0.0063, nugget = 0, sill = 0.0063 and the practical range (defined as the distance at which the variogram value was 95% of the sill) corresponded to approximately 2.4 km at the equator. The summary of the residual semivariogram (Figure 2b) showed partial sill = 0.1051, nugget = 0.8897, sill = 0.9948 and the practical range was approximately 3.2 km at the equator (Table 1). The residual semivariogram showed that 10.56% of the variation due to spatial aggregation.

### 2.2. Farm Level and Socio-Environmental Risk Factors Associated with C. burnetii Seroprevalence

The best-fitting non-spatial multivariable model included the farm-level variable of herd size and the environmental variables of population density, road density, elevation and three landcover classes (Table 2). Our results indicated that while herd size, elevation and the three landcover classes (i.e., grassland, cropland and built areas) were positively associated with *C. burnetii* seropositivity, population density and road density showed a negative association with *C. burnetii* prevalence. However, none of the associations were statistically significant.

The results of our Bayesian binomial geostatistical model indicated that, after accounting for the spatial dependence between surveyed farms, the *C. burnetii* seroprevalence had a significant negative association with the underlying human population density. Our results also indicated that, while not statistically significant, the variables elevation, tree cover and road density were positively associated with *C. burnetii* seroprevalence, and that cropland and built-up areas were negatively associated with *C. burnetii* seroprevalence (Table 3).

Our geostatistical model also indicated that after accounting for farm-level and socio-environmental predictors and spatial autocorrelation, the radii of *C. burnetii* seroprevalence in Jimma town was 6.9 km.

### 2.3. Predictive Distribution of C. burnetii Seroprevalence

**Figure 3:**
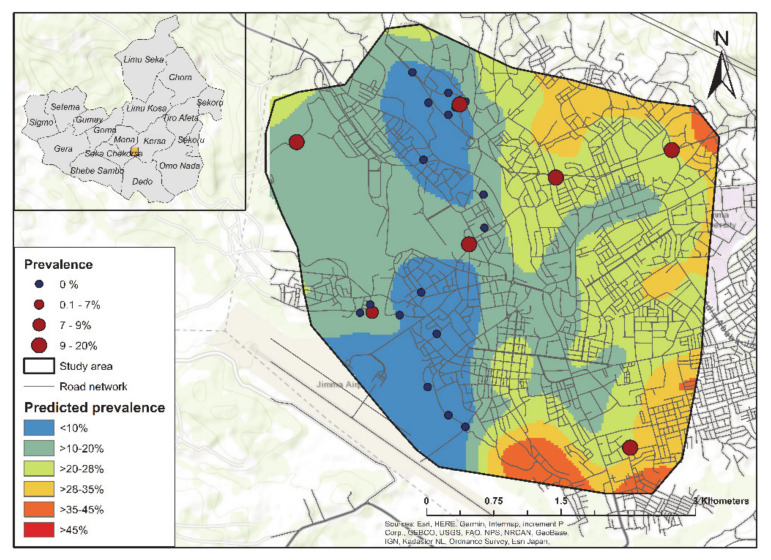
Predicted spatial distribution of *C. burnetii* seroprevalence based on the mean predicted values for the 25 dairy cattle examined in Jimma town.

Our predictive *C. burnetii* seroprevalence distribution map (Figure 3) indicated significant spatial heterogeneity and particularly high exposure risk in the eastern area of Jimma town (>20–45%). The predicted seroprevalence was higher (>10–20%) in two clusters in the north-west and south-west of Jimma town compared to the central and western areas of the town (<10%).

## 3. Discussion

Our model-based geographical study showed that the *C. burnetii* exposure risk from dairy cattle varied significantly across the landscape in Jimma town, Oromia Region, Ethiopia. Evidence of clustering of *C. burnetii* seropositivity in peri-urban and urban herds was found, and *C. burnetii* seroprevalence was significantly associated with a lower population density. Together these results indicated a significant risk to communities in the vicinity of these infected farms, which needs to be further investigated. Indeed, our results demonstrated that the predicted risk of community *C. burnetii* exposure to potentially infected dairy cattle was estimated to be the highest in the eastern parts of Jimma town, providing an opportunity for further investigations into seroprevalence in the affected communities. 

The finding that *C. burnetii* seroprevalence was clustered in Jimma town up to a distance of 800 m indicated a potential dispersal pattern, which is consistent with previous Q fever studies in urban settings [20,21]. Our result is in line with that of an urban outbreak study from Germany in 2005, where the attack rate was 1.3% at 350–400 m [22]. In our study, this distance was slightly larger than that previously described and this difference could be associated with factors that affect geographical dispersion, such as wind speed, barriers in the landscape [12] and cattle movement relationships between farms; unfortunately, we did not have such data to include in the model. 

Our results of the residual spatial dependence in *C. burnetii* seroprevalence after accounting for farm-level and environmental risk factors suggested that clustering *C. burnetii* remains unexplained by other farm-level variables that were not included in our non-spatial multivariable model. Important farm-level variables that should be considered in further investigations include information about biosecurity measures that were implemented in each farm, the introduction of new animals into the herd and relationships with Q fever infection areas (e.g., synchronicity with other ruminant outbreaks in Jimma zone) [12]. Furthermore, there could be important small-scale ecological factors, the influence of socio-economic factors, environmental bacterial contamination and bacterial survival that should be taken into account to estimate the risk of *C. burnetii* exposure [23]. For example, the environmental variables of wind speed and direction could also be considered in future studies since *C. burnetii* attaches to dust particles, which suggests a prominent role of wind-borne dispersal [9,10].

Due to the estimated presence of unexplained residual spatial clustering, a geostatistical model was implemented to account for the residual spatial clustering across Jimma town and predict *C. burnetii* at unsampled locations across the town. Our results indicated that the risk of *C. burnetii* exposure was higher in areas with a lower population density, which is consistent with the fact that cattle farms are normally located in peri-urbanised areas with a lower population density. Furthermore, this association is consistent with a previous study of an outbreak in Germany, where it was suggested that the urbanisation of rural areas was a potential factor that contributed to *C. burnetii* outbreaks [24]. Together, these findings have important public health implications. A preventive measure to avoid *C. burnetii* exposures that was adopted by some countries is based on regulating the proximity of urban residential areas to livestock facilities. In Germany, these urban planning regulations involve a 500 m residential construction exclusion area around sheep farms [25]. Meanwhile, in Australia (according to the Communicable Diseases Network Australia), their recommendation of a 1 km residential exclusion from abattoirs is based on the Netherlands outbreak [26]. 

Our predictive *C. burnetii* seroprevalence map indicated that predictive risk was not constant in Jimma town and there were areas, particularly to the east of the town, where the risk of *C. burnetii* exposure was predicted to be higher. While our previous study demonstrated that cattle in Jimma town were under a high level of *C. burnetii* exposure [15], our study expanded current knowledge in that it demonstrated that the east area of the town presented the higher predicted seroprevalence compared to other parts of the town. Our seroprevalence prediction map might help with targeting resources to ensure that communities that are most at risk of Q fever infection to the east of Jimma town are surveyed. Moreover, our predictive *C. burnetii* seroprevalence distribution map is an important decision support tool to target not only livestock Q fever management plans but also the deployment of subsequent follow-up studies to the human communities within the identified high-risk areas.

The findings of this study need to be interpreted in light of some important limitations. First, the small sample size of the cattle herd samples and the small study area (approx. 54 km^2^) were limiting factors regarding drawing robust conclusions about the generalisability of the results to other areas in the Jimma zone. Moreover, given the high level of livestock production in the Jimma zone, other areas are also possibly at risk. Second, although we found a significant association with human population density, it has to be taken into account that this result is based on the resolution of the available human population density raster, which was limited to a 5 km grid. Finally, while we found a higher risk of *C. burnetii* seroprevalence to be associated with some ecological variables, elevation, tree cover and road density (as a proxy of the density of residential areas), these effects were not statistically significant. These results are not unexpected in an ecological study, as effects will tend to the null; this is known as regression dilution bias [27]. 

In conclusion, our findings indicated that *C. burnetii* seroprevalence in dairy cattle farms was highly clustered in Jimma town and that the geographical clustering was associated with the contextual factors of the community. This study could also be used as a model for other countries worldwide. Our results can form the basis for a decision support tool to guide further empirical studies in livestock and people in the eastern part of Jimma town where the risk of dairy cattle exposure to *C. burnetii* was predicted to be the highest.

## 4. Materials and Methods

### 4.1. Sampling and Environmental Data Collection

To estimate the seroprevalence of *C. burnetii* infection in the Jimma dairy herds, a total of 227 cattle were tested in 25 dairy farms between October 2016 and October 2017 [15]. Blood samples were collected from the jugular vein from the 227 animals using a vacutainer tube [see reference [15] for more details]. A total of 14 animals were seropositive for *C. burnetii* [15]. The highest farm-level seroprevalence was 20% and serum samples were tested using Indirect Enzyme-Linked Immunosorbent Assay (i-ELISA) from ID Screen^®^ Q Fever Indirect Multi-Species kits (ID.vet, Grabels, France) for the detection of antibodies against *C. burnetii* [15]. The overall seroprevalence for each farm was calculated and included in a map for visualisation purposes. The location of each farm was obtained from the livestock and fisheries resources development office in Jimma town [15] and the Jimma town dairy cooperative office. The coordinates (longitude and latitude) data of each farm was collected using the Garmin GPS map 64 series. 

We obtained a set of socio-environmental variables to explain the geographical variation in *C. burnetii* exposure in dairy cattle in Jimma town. *C. burnetii* can survive in the environment and therefore environmental variables can be important predictors of exposure. In fact, *C. burnetii* can attach to dust particles [9,10]; hence, windborne spread has been linked with Q fever outbreaks [28]. Higher elevation is associated with stronger winds [29]; therefore, elevation was considered in our analyses. Open areas can influence the dispersal of dust particles [30] and therefore was considered as an environmental predictor. Population density and road density were considered to investigate the public health risk that was associated with dairy cattle seropositivity. 

Elevations were obtained from the ASTER Global Digital Elevation Model (GDEM) Version 3 (ASTGTM). Land cover was obtained from the ESA Land Cover CCI – S2 prototype LC map of Africa 2016 at a 20 m resolution. The land cover categories that overlapped with the study area were: tree cover areas, shrubs cover areas, grassland, cropland, vegetation aquatic, bare areas, built areas and open water. For the prediction analysis, the land cover types were reclassified into three categories: tree cover, cropland and built areas. Grassland was included as a reference, as well as the rest of the land cover categories. A raster map that contained the human population density in 2000 (GPW V3, UN Adjusted) with a 2.5 arc-minutes resolution was obtained from CIESIN. The road density was used as a proxy of the density of residential areas in Jimma town and was calculated using the line density tool that is available in the ArcGIS (version 10.8) toolbox (Table 4). All environmental variables were downloaded in December 2020. Pearson’s correlation coefficients were used to explore the correlations between environmental variables.

### 4.2. Analysis of Clustering

To investigate geographical clustering in *C. burnetii* seropositivity in Jimma town, Ethiopia, semivariograms were used. The extent of the geographical clustering in Coxiellosis was quantified by using a semivariogram, which showed the average cluster size of *C. burnetii* seropositive farms and how strong the clusters were. A semivariogram is a plot of the semivariance of all pairs of positions under a series of defined separation distances and can be defined by three parameters, namely the nugget, the range and the sill. The sill is constituted by the sum of the partial sill and the nugget. The partial sill and nugget correspond, respectively, to the components of the residual variation that are spatially structured and unstructured (e.g., random error) variations. The nugget can represent natural random changes, very small-scale spatial variation or measurement errors. The range indicated the average size of clusters of *C. burnetii* seroprevalence. The residuals of the non-spatial model were also investigated for spatial autocorrelation to estimate how much spatial variation in *C. burnetii* seroprevalence was explained by the variables in the non-spatial model. The semivariogram was constructed using the *geoR* package and the model parameters were estimated using weighted least squares [31] in R [32]. 

### 4.3. Risk Factors Associated with Farm-Level Seroprevalence

We used a binomial generalised linear model to quantify associations between the farm-level *C. burnetii* seroprevalence and the farm-level biosecurity risk factors and socio-environmental predictors. The statistical analysis was performed in two steps. First, we performed univariable analysis to select variables to be included in the final multivariable model based on a *p*-value < 0.25. Second, we performed multivariable analysis by including the previously selected variables and all the ecological variables for the final multivariable model. Models were performed using Stata version 15.1 software [33].

### 4.4. Modelling the Geographical Risk Prediction of C. burnetii Seroprevalence

Based on the results of the residual semivariogram analysis, we decided to implement a Bayesian geostatistical binomial model to account for the residual spatial autocorrelation. The model was adjusted for data on socio-environmental risk factors correlated with the prevalence of *C. burnetii* seropositivity in Jimma, Ethiopia, by using OpenBugs version 3.2.3 (MRC Biostatistics Unit, Cambridge, and Imperial College London, U.K.). The model included intercept and environmental variables, such as elevation, land cover, population density and road density. The numbers of tested and positive animals were included as observed data. A total of 25 individual observations of *C. burnetii* status and 4736 individual prediction points were considered in the analysis, where the points were distributed in a grid that overlapped the study area such that adjacent points were separated by 110 m across Jimma’s landscape. The mean predicted values were used to create a predicted spatial distribution of *C. burnetii* seroprevalence using a kernel function, which calculates a magnitude per-unit area from points in ArcGIS desktop version 10.8. The outputs of Bayesian models, including parameter estimates and spatial prediction at unsampled locations, are distributions termed “posterior distributions”. The posterior distributions fully represent the uncertainties associated with the parameter estimates. The full model specification is included in Supplementary File S1. The parameter phi (φ) indicates the size of clusters and refers to the rate of decay of the spatial autocorrelation; the radius of the cluster corresponded to (3/φ) × 111 (one decimal degree is equivalent to approximately 111 km at the equator).

## Figures and Tables

**Table 1:** Results from the variogram-based parameter estimations using the weighted-least-squares method for the raw data and the residuals.

Parameters	Raw Data	Residuals
Range (φ)	0.0072 (~0.8 km)	0.0096 (~1.1 km)
Nugget (τ^2^)	0	0.8897
Partial sill (σ^2^)	0.0063	0.1051
Sill	0.0063	0.9948
Variance of spatial random effect	0%	10.56%

**Table 2:** Univariable and multivariable binomial generalised linear models at the farm level (*n* = 25) for *C. burnetii* in dairy cattle herds of Jimma town.

Variable	Univariable Analysis			Best-Fitting Multivariable Analysis		
	Coef.	95% CI	*p*-Value	Coef.	95% CI	*p*-Value
***Farm-Level Factors***						
Multi-age mix (ref. yes)	0.28	(−0.81, 1.37)	0.62			
Multi-species mix (ref. yes)	16.04	(−4449.44, 4481.51)	0.99			
Tick infestation (ref. yes)	0.18	(−0.9, 1.26)	0.75			
Herd size (continuous) *	0.01	(0, 0.03)	0.17	0.01	(−0.14, 0.34)	0.42
Connected with other herd (ref. yes)	0.53	(−1.04, 2.1)	0.51			
Management system: semi-intensive (ref. intensive)	0.58	(−0.51, 1.67)	0.30			
Nuisance (ref. yes)	0.45	(−0.86, 1.76)	0.50			
Abortion history in the herd (ref. yes)	−0.48	(−2.04, 1.09)	0.55			
***Ecological Factors***						
Elevation *	0.02	(−0.01, 0.06)	0.21	0.001	(−0.001, 0.004)	0.32
Population density *	−0.01	(−0.02, 0)	0.20	−0.01	(−0.07, 0.06)	0.89
Road density	0.0002	(0, 0)	0.73	−0.01	(−0.03, 0.01)	0.16
Landcover: grassland (ref. tree cover)	−0.74	(−2.89, 1.4)	0.50	4.76	(−11.01, 20.52)	0.55
Landcover: cropland (ref. tree cover)	−0.78	(−2.39, 0.83)	0.34	4.64	(−11.79, 21.06)	0.58
Landcover: built areas (ref. tree cover)	−0.26	(−1.53, 1.02)	0.70	3.56	(−12.28, 19.4)	0.66

* Variables with *p*-value < 0.25; Coef: regression coefficient; CI: confidence interval; ref: reference.

**Table 3:** Estimates of model parameters for *C. burnetii* seroprevalence based on a Bayesian geostatistical logistic regression model.

Variable	Posterior Mean	2.5CrI	97.5CrI
Elevation	0.014	−0.08	0.16
Landcover: tree cover (ref. grass cover)	1.48	−7.14	7.82
Landcover: cropland (ref. grass cover)	−0.72	−6.76	4.49
Landcover: built area (ref. grass cover)	−1.65	−7.13	3.11
Population density *	−0.02	−0.04	−0.002
Road density	0.001	−0.003	0.004
Intercept	−24.01	−274.8	136.6
*Spatial Effect*			
Phi (φ)	48.3	2.70	97.35
Tau (τ)	61.07	0.01	541.5

* Significant effect; CrI: credible interval; ref: reference.

**Table 4:** Environmental variables included in the model for the *C. burnetii* geographical risk prediction to the population of Jimma town.

Environmental Variable	Data Source	Spatial Resolution
Elevation (metres)	ASTER Global Digital Elevation Model (GDEM) Version 3 (ASTGTM) (https://earthdata.nasa.gov, accessed on 1 December 2020)	30 m grid cell
Land cover classifications	ESA Land Cover CCI – S2 prototype LC map of Africa 2016 (http://2016africalandcover20m.esrin.esa.int/, accessed on 1 December 2020)	20 m grid cell
Human population density (number of people per 5 km^2^)	GPW V3, UN-adjusted, CIESIN (http://sedac.ciesin.columbia.edu/data/set/gpw-v3-population-density, accessed on 1 December 2020)	~5 km grid cells
Road network (used to calculate the road density)	Ethiopia GIS Dataset (https://open.africa/dataset/ethiopia-gis-dataset, accessed on 1 December 2020)	-

## Data Availability

The data supporting the findings of the article are not available publicly due to ethical reasons and are available from the corresponding author upon reasonable request.

## Supplementary Materials

The following are available online at https://www.mdpi.com/article/10.3390/pathogens10060646/s1, Supplementary File S1 contains the statistical notation of Bayesian geostatistical models and spatial interpolation. Reference [34] is cited in the supplementary materials.
