# Supplementary material for: Geographical Variation in Coxiella burnetii Seroprevalence in Dairy Farms Located in South-Western Ethiopia: Understanding the Broader Community Risk
Source: Pathogens. 2021 May 23;10(6):646. doi: 10.3390/pathogens10060646 (PMC8224686; doi:10.3390/pathogens10060646)
Supplement: Supplementary file 1 [file pathogens-10-00646-s001.zip › pathogens-1181901-supplementary.pdf]

Supplementary File 1. Statistical notation of Bayesian geostatistical models for seroprevalence of *C. burnetii* in Jimma town, Ethiopia.

The number of positive animals to *C. burnetii* antibodies in each farms *j*, is a binomial variable Pos_j, and the number of individuals tested in each farm is $Test_j$. The model assumes a conditional binomial model where the seroprevalence of *C. bunertii* n_j is given by:

$$Pos_j \sim Binomial(n_j, Test_j) \quad (1)$$

The mean seroprevalence of *C. burnetii* antibodies in each farm *j*, was modelled by:

$$logit(p_j) = \alpha + \sum_{z=1}^{Z} \beta \times x_j + u_j \quad (2)$$

In the model, α represent the intercept, β is a matrix of coefficient while x is a matrix of covariate and u_j are coefficients representing geostatistical random effects. These random effects have a multinomial normal distribution, of mean zero and variance-covariance matrices defined by anisotropic powered exponential spatial correlation function:

$$f(d_{ab}; \phi) = \exp[-(\phi d_{ab})] \quad (3)$$

where d_{ab} are the distances between pairs of points *a* and *b*, and ϕ is the rate of decline of spatial correlation per unit of distance. Non-informative priors were used for α (uniform prior with bounds -∞ and ∞) and the coefficients (normal prior with mean = 0 and precision = 1 × 10^{-4}). The prior distribution of ϕ was also uniform with upper and lower bounds set at 0.1 and 100. (the lower bound set to ensure spatial correlation at the maximum separating distance between farms locations was <0.5, assisting identifiability [34]. The precision of u_j was given a non-informative gamma distribution.

A burn-in of 5,000 iterations was allowed, followed by 10,000 iterations. Intercept, coefficients, and predicted probability values of seroprevalence at the prediction locations were stored. Visual examination of history and density plots was undertaken to check for convergence of the stored variables. Convergence was successfully achieved after 5,000 iterations.

The predictions of the seroprevalence of *C. burnetii* in Jimma town were made at the nodes of a 0.001 x 0.001 decimal degree grid (approximately 110m^2) by interpolating the geostatistical random effect and adding it to the sum of the products of the coefficients for the fixed effects and the values of the fixed effects at each prediction location.

The *spatial.unipred* function, which implements Bayesian kriging in OpenBUGS, was used for the interpolation of the random effect. The values of predicted prevalence at unsampled locations are estimated independently of neighbouring values.

The outputs of Bayesian models, including parameter estimates and spatial prediction at unsampled locations, are distributions termed "posterior distributions". The posterior distributions fully represent uncertainties associated with the parameter estimates. The posterior distributions were summarised in terms of the posterior mean and 95% Bayesian credible interval (CrI), within which the true value occurs with a probability of 95%.

The variables were standardised by subtracting the mean and dividing by the standard deviation. The resulting regression coefficients for these variables represent the change in terms of standard deviations in prevalence that result from a change of one standard deviation in these variables.
